# 异基因造血干细胞移植治疗经典型阵发性睡眠性血红蛋白尿症5例临床观察

**DOI:** 10.3760/cma.cn121090-20240612-00221

**Published:** 2024-12

**Authors:** 小利 赵, 利宁 张, 潇予 张, 梦泽 郝, 书连 陈, 嘉璘 魏, 祎 何, 明哲 韩, 尔烈 姜

**Affiliations:** 1 中国医学科学院血液病医院（中国医学科学院血液学研究所），实验血液学国家重点实验室，国家血液系统疾病临床医学研究中心，细胞生态海河实验室，天津 300020 State Key Laboratory of Experimental Hematology, National Clinical Research Center for Blood Diseases, Haihe Laboratory of Cell Ecosystem, Institute of Hematology & Blood Diseases Hospital, Chinese Academy of Medical Sciences & Peking Union Medical College, Tianjin 300020, China; 2 天津医学健康研究院，天津 301600 Tianjin Institutes of Health Science, Tianjin 301600, China

## Abstract

2019年至2023年期间，5例经典型阵发性睡眠性血红蛋白尿症（cPNH）患者在中国医学科学院血液病医院接受异基因造血干细胞移植。5例患者均为男性，中位年龄26（26～46）岁，诊断到移植的中位间隔时间为5.5（3.6～18.0）年，移植前粒细胞中位PNH克隆为96.3％（90.0％～99.7）％，中位乳酸脱氢酶2 224（1 244～4 753）U/L，骨髓增生活跃至极度活跃。单倍体移植4例，同胞全相合移植1例。清髓性预处理（MAC）3例，减低剂量预处理（RIC）2例。未发生原发性植入失败及移植后早期死亡。中位中性粒细胞植入时间为15（13～21）d，中位血小板植入时间为24（13～60）d。3例患者移植后发生Ⅱ度急性移植物抗宿主病（GVHD），2例患者发生局限型慢性GVHD。5例患者移植后PNH克隆均转阴，中位转阴时间为移植后2（1～3）个月。中位随访16（6～34）个月，1例患者死于疾病复发合并感染。存活的4例患者中，2例已停用所有药物，1例口服环孢素A治疗皮肤慢性GVHD，另外1例口服环孢素A常规预防GVHD。

## Abstract (en)

This study enrolled five patients with classic paroxysmal nocturnal hemoglobinuria（cPNH）who underwent allogeneic hematopoietic stem cell transplantation in our hospital from 2019 to 2023. All five patients were male, with a median age of 26（range: 26–46) years. The median time from diagnosis to allo-HSCT was 5.5（range: 3.6–18.0）years. The median PNH granulocyte clone size was 96.3％（ranged 90.0％–99.7％）, and the median lactate dehydrogenase（LDH）level was 2 224 IU/L（8896-fold of the upper limit of normal）. All patients were detected to have bone marrow hyperplasia by trephine biopsy. The stem cell source came from four haploidentical donors and one HLA-identical sibling donor. Among the five patients, three underwent myeloablative conditioning（MAC）and two underwent reduced-intensity conditioning（RIC）treatment. None of the patients experienced primary implantation failure. The neutrophil implantation time was 15（range: 13–21）days, and the platelet implantation time was 24（range: 13–60）days. The three patients developed grade II acute graft-versus-host disease（aGVHD）. No patients developed grade Ⅲ/Ⅳ aGVHD. The two patients developed localized chronic GVHD（cGVHD）, and no patients developed extensive cGVHD. PNH clones turned negative in all patients after 2（range: 1–3）months of transplantation. At a median follow-up of 16（range: 6–34）months, one patient died of relapse and infection, and the remaining four patients survived, of which two patients had discontinued all drugs.

阵发性睡眠性血红蛋白尿症（PNH）是一种罕见的PIG-A突变导致的造血干细胞获得性克隆性疾病。补体旁路途径的持续活化在临床过程中发挥重要作用。随着补体单克隆抗体的出现，PNH的预后获得极大的改善。C5单抗不仅能够有效减轻溶血，同时减少血栓事件的发生，是目前经典型PNH（cPNH）的主要治疗手段[1]–[2]。但是，一方面部分国家、地区不能获得该类药物，另一方面，即使行补体抗体治疗，PNH克隆不能被清除，部分患者仍输血依赖[3]，同时长期服药伴随极大的经济负担。对于cPNH的患者，如果不能获得补体抗体药物并且经其他药物治疗后仍输血依赖或者反复发生危及生命的血栓，或补体抗体药物疗效欠佳，或有脱离口服药物治疗需求，异基因造血干细胞移植（allo-HSCT）仍然是该类患者的选择。由于PNH发病率较低，既往研究纳入病例时间跨度长，并且很少单独探讨allo-HSCT治疗cPNH。本研究纳入5例近年来在我中心接受allo-HSCT的cPNH患者，评估allo-HSCT对于cPNH的疗效。

## 资料与方法

1. 病例及随访：纳入我院2019年至2023年行allo-HSCT治疗的5例cPNH患者，诊断标准参照国际PNH工作组制定的临床分型[4]。5例患者均为糖皮质激素联合环孢素A或单独糖皮质激素治疗效果欠佳且不能获得C5单抗类药物。

2. 预处理方案：5例患者均采用白消安（Bu）/环磷酰胺（Cy）为基础的预处理方案。其中，3例患者行3天Bu方案的清髓性预处理（MAC），2例患者行2天Bu方案的减低剂量预处理（RIC）。

3. 移植后主要并发症防治：采用他克莫司或环孢素A联合短疗程甲氨蝶呤±芦可替尼（5 mg每日2次）±霉酚酸酯预防移植物抗宿主病（GVHD）。所有患者均入住百级层流病房，移植前予复方磺胺甲噁唑（1.92 g/d × 5 d）预防卡氏肺孢菌肺炎；予阿苯达唑0.4 g清除寄生虫。所有患者移植后均给予泊沙康唑预防真菌感染、阿昔洛韦预防单纯疱疹病毒感染以及复方磺胺甲噁唑预防卡氏肺孢菌肺炎。

4. 指标监测：入仓前及移植后定期嗜水气单胞菌溶素变异体（Flaer）流式细胞术检测外周血红细胞、粒细胞及单核细胞GPI蛋白，监测患者PNH克隆。

中性粒细胞绝对计数（ANC）≥0.5×10^9^/L连续3 d的第一天为粒细胞植入日期；脱离血小板输注且连续7 d血小板计数≥20×10^9^/L的第一天为血小板植入日期。以短串联重复序列（STR）、性染色体荧光原位杂交判定嵌合状态。完全供者嵌合体指嵌合体中供者成分>95％，混合嵌合体指嵌合体中供者成分占5％～95％。原发性植入失败定义为移植后28 d包括ANC<0.5×10^9^/L的全血细胞减少且至少2次骨髓未检出供者成分。

5. 随访：患者生存相关资料来自住院/门诊病历和电话随访记录。移植后中位随访时间为16（6～34）个月。

## 结果

1. 临床特征：5例患者均为男性，中位年龄26（26～46）岁，以间断血红蛋白尿起病，无血栓形成病史，移植前均有甲泼尼龙治疗史，其中2例联合环孢素A治疗。移植前粒细胞中位PNH克隆96.3（90.0～99.7）％，中位乳酸脱氢酶为2 224（1 244～4 753）U/L，骨髓活检示骨髓增生活跃至极度活跃。诊断至移植的中位间隔时间为5.5（3.6～18）年。4例患者行单倍体移植，1例行同胞全相合移植。2例患者胆红素升高，造血干细胞移植共患病指数（HCT-CI）评分1分。单个核细胞输注量为13.36（9.74～22.00）×10^8^/kg，CD34^+^细胞输注量为3.26（1.98～5.75）×10^6^/kg。患者起病时及移植前临床资料见表1、表2，预处理方案见表3。

**表1:** 5例经典型阵发性睡眠性血红蛋白尿症（cPNH）患者起病时临床特征

例号	性别	年龄（岁）	WBC（×10^9^/L）	HGB（g/L）	PLT（×10^9^/L）	RET（×10^12^/L）	PNH-红（%）	PNH-粒（%）	PNH-单（%）	LDH（U/L）
1	男	46	4.80	56	115	0.24	49.7	81.0	NA	2 636
2	男	26	4.60	66	97	0.24	29.0	81.0	76.0	1 569
3	男	43	5.11	51	180	0.31	49.8	87.5	NA	2 224
4	男	26	1.86	51	74	0.07	33.7	53.0	NA	1 027
5	男	26	5.80	51	136	0.27	49.7	90.8	90.3	1 451

**注** RET：网织红细胞；NA：缺失

**表2:** 5例经典型阵发性睡眠性血红蛋白尿症（cPNH）患者异基因造血干细胞移植前临床特征

例号	性别	年龄（岁）	WBC（×10^9^/L）	HGB（g/L）	PLT（×10^9^/L）	RET（×10^12^/L）	PNH-红（%）	PNH-粒（%）	PNH-单（%）	LDH（U/L）	骨髓增生
1	男	46	0.61	74	16	0.22	38.1	99.7	99.7	4 753	活跃
2	男	26	6.05	33	135	0.24	55.0	96.3	95.1	2 332	明显活跃
3	男	43	4.76	66	343	0.33	98.6	98.7	98.1	2 224	活跃
4	男	26	4.40	76	142	0.22	82.8	91.7	94.3	2 057	活跃
5	男	26	7.14	68	143	0.27	70.9	90.0	91.0	1 244	明显活跃

**注** RET：网织红细胞；NA：缺失

**表3:** 5例经典型阵发性睡眠性血红蛋白尿症（cPNH）患者异基因造血干细胞移植预处理方案

例号	移植类型	预处理方案	具体药物
1	MSD	MAC	Bu 3.2 mg·kg^−1^·d^−1^× 3 d，Flu 30 mg·m^−2^·d^−1^× 3 d，Cy 40 mg·kg^−1^·d^−1^× 2 d，rATG 2.5 mg·kg^−1^·d^−1^× 3 d
2	haplo	MAC	Bu 3.2 mg·kg^−1^·d^−1^× 3 d，Cy 40 mg·kg^−1^·d^−1^× 4 d，rATG 2.5 mg·kg^−1^·d^−1^× 5 d
3	haplo	RIC	Bu 3.2 mg·kg^−1^·d^−1^× 2 d，Flu 30 mg·m^−2^·d^−1^× 5 d，Cy 40 mg·kg^−1^·d^−1^× 4 d，rATG 2.5 mg·kg^−1^·d^−1^× 4 d
4	haplo	RIC	Bu 3.2 mg·kg^−1^·d^−1^× 2 d，Flu 30 mg·m^−2^·d^−1^× 5 d，Cy 40 mg·kg^−1^·d^−1^× 4 d，rATG 2.5 mg·kg^−1^·d^−1^× 4 d
5	haplo	MAC	Bu 3.2 mg·kg^−1^·d^−1^× 3 d，Flu 30 mg·m^−2^·d^−1^× 5 d，Cy 40 mg·kg^−1^·d^−1^× 3 d，rATG 2.5 mg·kg^−1^·d^−1^× 4 d

**注** MSD：同胞全相合移植；haplo：单倍体移植；MAC：清髓性预处理；RIC：减低强度预处理；Bu：白消安；Flu：氟达拉滨；Cy：环磷酰胺；rATG：兔抗人胸腺细胞免疫球蛋白

2. 造血重建：所有患者均未发生原发性植入失败、移植物功能不良及移植后早期死亡。中位中性粒细胞植入时间为15（13～21）d，中位血小板植入时间为24（13～60）d。植入后1例患者于移植后3个月因疾病复发发生供者嵌合度下降，出现混合嵌合状态伴血细胞三系减少，余4例均供者嵌合状态且脱离输血及造血生长因子支持治疗。

3. 主要并发症：植入前2例患者分别发生2、3度口腔黏膜炎，2例患者分别出现胃肠炎、肛周软组织感染，粒细胞植入后，以上临床症状均获得完全缓解。植入后，3例患者发生巨细胞病毒血症，中位发生时间为30（26～40）d，经更昔洛韦或膦钾酸钠抗病毒治疗后转阴。1例于移植后45 d诊断EB病毒血症，经利妥昔单抗治疗后转阴。移植后4例患者发生不同部位细菌感染（口腔、肺、血液、泌尿系），中位发生时间为移植后2（0.9～5）个月，其中1例因耐药铜绿假单胞菌败血症死亡。4例患者发生1～3级出血性膀胱炎，中位发生时间为移植后26（15～34）d，经水化、碱化、膀胱冲洗等治疗后缓解。3例患者发生Ⅱ度急性GVHD，中位发生时间为移植后26（24～45）d，1例加用甲泼尼龙治疗后缓解，另外2例联合芦可替尼、间充质细胞治疗后完全缓解。未发生发生Ⅲ/Ⅳ度急性GVHD。所有患者移植后生存均超过100 d，2例发生慢性GVHD。其中1例局限于肺，目前随访34个月，已停用所有药物治疗；1例局限于皮肤，目前随访16个月，仍口服环孢素A治疗，病情稳定。

4. 预后：移植后每月检测一次外周血PNH克隆，5例患者移植后PNH克隆均转阴，时间为回输后2（1～3）个月。至随访时间1例患者因复发、感染死亡，余4例患者存活，其中2例已停用所有药物，1例仍口服环孢素A治疗皮肤慢性GVHD，1例环孢素A常规预防GVHD。例1自移植后3个月STR开始下降，同时伴随全血细胞减少及PNH克隆复发，逐步减停环孢素A，并于移植后3.6个月予以供者淋巴细胞输注，但STR继续下降、PNH克隆继续上升。于移植后4.5个月予以FLAG方案化疗及供者淋巴细胞输注，患者于骨髓抑制期死于耐药铜绿假单胞菌败血症合并多脏器功能不全，未评估疗效。

## 讨论

对于存在骨髓衰竭、克隆演变、不能获得补体抗体药物或者有脱离长期药物治疗需求及补体抗体疗效欠佳的患者，allo-HSCT仍是唯一的治疗选择[5]–[7]。在不能获得补体抗体的情况下，难治性PNH、反复出现危及生命的血栓栓塞事件也可作为allo-HSCT指征。同时，随着造血干细胞移植技术的进步，近年的PNH移植成功率也在逐步提高[8]。

由于发病率低以及补体抗体药物的应用，关于allo-HSCT治疗PNH的报道相对较少。另外，大多数cPNH患者骨髓增生活跃，诊断前病程较长[9]，血管内存在大量游离血红蛋白而导致内皮系统脆弱[10]。多数研究没有单独探讨cPNH患者中allo-HSCT的疗效及安全性。

在供者的选择方面，在干细胞来源涉及单倍体供者、全相合同胞供者、全相合无关供者以及脐血干细胞的多项研究中，未发现干细胞来源对PHN患者的移植疗效具有影响[9],[11]–[17]。本研究中，4例患者行单倍体移植且预后良好，提示单倍体移植可作为cPNH有效的治疗选择。

目前，cPNH患者无统一推荐的预处理方案。多项研究比较了MAC与RIC方案对预后的影响，认为RIC方案可以清除PNH克隆，预后不劣于MAC方案[8],[12],[14]。只有2010年意大利的一项研究发现RIC方案的非复发死亡率高于MAC方案，1年总生存率为47％[11]。研究组分析归因于RIC组非亲缘供者占比较高以及RIC方案种类不一[11]。近年的几项研究选择RIC方案，并且获得不错的疗效[9],[13],[15]。其中，2014年Pantin等[13]采用氟达拉滨（Flu）/Cy为基础的RIC方案，移植后6年总生存率达87.8％。基于cPNH患者骨髓增生活跃至极度活跃以及PNH克隆细胞免疫抗性[18]，我们在5例cPNH患者预处理方案中均加用了Bu，仅1例同胞全相合移植患者死亡，其余4例存活，提示RIC方案单倍体移植在cPNH患者中是可行的，当然MAC与RIC孰优孰劣尚需进一步扩大样本量验证。

植入失败、GVHD是PNH患者移植后主要面临的问题[9],[11],[15]。为了减少植入失败，本组患者采用Bu/Cy为基础的预处理方案，5例患者均获得成功植入。cPNH患者内皮系统受损、补体活化[10]，这两个病理生理过程与移植后早期并发症（GVHD、移植相关血栓微血管病和肝窦闭塞综合征）相关[19]。为减少内皮系统进一步损伤，同时考虑预处理治疗的免疫强度，我们将Cy总量定为120～150 mg/kg，偏低于其他研究报道的剂量[17]。为了预防GVHD，2例患者预处理过程中加入芦可替尼5 mg每日2次。本组5例患者均未发生重度急性GVHD及广泛型慢性GVHD，也未发生移植相关血栓微血管病。至随访截止，4例患者生存，并且其中2例已完全脱离药物治疗。Nakamura等[15]研究发现输血次数>20次、年龄≥30岁是影响PNH患者allo-HSCT的预后因素。本研究中死亡患者（例1）年龄46岁、输血>20次，同时伴发贫血性心脏病，预处理期间下调了Cy总量（40 mg/kg × 2 d），在同胞全相合移植后仍发生原发病复发，最后因感染死亡。

由于cPNH发病率低，本研究样本量小，随访时间较短，预处理方案的优化需进一步探讨与验证。另外，本研究为回顾性研究，受限于当时不能获得补体抗体药物，无相关对照资料进一步论证allo-HSCT治疗的优势。
